# Trends in and predictors of animal source food consumption among 6–23 months age children in Tigrai, Northern Ethiopia: evidence from three consecutive ethiopian demographic and health surveys, EDHS 2005–2016

**DOI:** 10.1186/s40795-023-00699-9

**Published:** 2023-03-08

**Authors:** Gebretsadkan Gebremedhin Gebretsadik, Zuriyash Tadesse, Tesfay Yohannes Ambese, Afework Mulugeta

**Affiliations:** 1 Department of Nutrition and Dietetics, School of Public Health, College of Health Sciences, Mekelle University, Mekelle, Ethiopia; 2 Department of Public Health Surveillance, Ethiopian Public Health Institute Tigrai Branch, Mekelle, Ethiopia

**Keywords:** ASF, Children, EDHS, Tigrai

## Abstract

**Background:**

Despite numerous interventions, child undernutrition continues as a problem of global concern. Although consumption of animal source foods has shown positive associations with child undernutrition, no much evidence exists on its trends and predictors among children in Tigrai.

**Objectives:**

This study aimed to investigate the trends in and predictors of consumption of animal source foods among children 6–23 months of age in Tigrai.

**Methodology:**

This study used complex data of 756 children extracted from three consecutive Ethiopian Demographic and Health Surveys. Data were analyzed using STATA 14.0 by accounting for sampling weight and cluster and strata variables. Multivariable logistic regression was used to determine the independent predictors of animal source foods consumption. Odds ratio and 95% confidence interval were used to measure strength of association at a statistical significance of p < 0.05.

**Results:**

Although statistically not significant (p-trend = 0.28), animal source foods consumption increased from 31.3% to 2005 through 35.9% in 2011 to 41.5% in 2016. For every month increase in the age of a child, a 9% increment in the odds of animal source food consumption was observed. Muslim children showed 3.1 times higher odds of animal source food consumption than Orthodox Christians. The likelihood of animal source foods consumption were 33% lower among children born to mothers who didn’t attend formal education as compared to their counterparts. A unit increase in the number of household assets and number of livestock led to a 20% and 2% increase in the odds of animal source foods consumption, respectively.

**Conclusion:**

Animal source foods consumption showed a statistically non-significant increase over the three consecutive Ethiopian Demographic and Health Surveys. This study found out that consumption of animal source foods might be increased through pro-maternal education policies, programs with household asset increasing schemes, and pro-livestock projects. Our study also highlighted the need for considering religion as one important player when planning or undertaking ASF programs.

## Background

Despite overwhelming number of interventions targeting it, child undernutrition continues to be a grave public health concern worldwide. In 2018, stunting and wasting affected an estimated 149 million (21.9%) and 49 million (7.3%) children under 5 globally, respectively [1]. Poor physical growth and cognitive development, lower school performance, morbidities, and about half of under-five deaths are attributed to child undernutrition [2–4]. It also has been associated with increased risk of obesity and chronic diseases later in life [5] .

Animal source foods (ASF) are calorie-dense foods that represent essential components of complementary foods [6]. Their essentiality vis-à-vis plant based foods lies on their capability to provide high quality proteins containing all the essential amino acids in adequate amounts and bioavailable micronutrients like iron, zinc, calcium, vitamin A, vitamin B12, and riboflavin [7, 8]. Out of the eight food groups used by the World Health Organization (WHO) to determine optimal dietary diversity in older infants and young children, three (flesh foods, egg, and dairy) are ASFs [9].

Optimal child complementary feeding practices are known to improve nutrition outcomes by increasing energy and nutrient intakes [10]. ASFs are known as key drivers of improved nutrition during early years of children’s lives, especially for those in low and middle income countries (LMICs) [11]. Although consumption of ASFs has recently been in a controversy over the risk of non-communicable diseases [12, 13] and environmental impacts related to their production such as greenhouse gas emissions [14], their benefits for physical and cognitive development remain substantial [15–17] especially in vulnerable groups from LMICs.

According to a recent report, in 2018, the daily average global consumption of unprocessed red meat among children was 40 g (95% UI 38–43), with regional differences varying from a highest 93 g in Central or Eastern Europe and Central Asia to a lowest 7 g in South Asia [18]. Few studies from Ethiopia has reported strong evidences on the relationship between consumption of ASFs – especially milk – and improved child nutritional outcomes [19, 20]. However, the diets of children are mostly monotonous and ASFs are rarely consumed. The proportion of 6–23 months old children consuming meat, fish, or poultry (MFP), eggs, and dairy was reported to be 8%, 17%, and 25%, respectively [21]. Besides, according to a study conducted in Tigrai region, only 13% of children aged 6–23 months meet the minimum dietary diversity score set by WHO [22]. Moreover, a recent study by Gebretsadik et al., reported a 46.5% magnitude of ASF consumption varying regionally from a lowest (20.2%) in Amhara region to a highest (78.2%) in Addis Ababa [23].

Based on the Ethiopian Demographic and Health Surveys (EDHS) report, Ethiopia has achieved a 14% reduction in child stunting rate over the past 15 years, although 37% of under 5 children remain still stunted [24]. Similarly, anemia is prevalent in 57% of children under five [21]. Zinc and vitamin A deficiencies are also problems of public health concern in children and mothers in Ethiopia [21, 24]. In Tigrai region, the prevalence of child stunting is higher than the national prevalence (49% vs. 37%) [24]. As means to tackle such problems of undernutrition, the country has been implementing the National Nutrition Program [25] recently endorsed a Food and Nutrition Policy [26] and applied a National Food and Nutrition Strategy [27].

Realizing the well established association between increased consumption of ASFs and better child nutritional outcomes, one main factor for the highest rates of child undernutrition in Tigrai could be low consumption of ASFs. However, there is no much evidence on the trends in consumption of ASFs from 2005 to 2016 and on predictors of consumption of ASFs among young children from Tigrai. Therefore, the aim of this study was to investigate the trends in and predictors of consumption of ASFs among children 6–23 months of age in Tigrai.

## Materials and methods

### Data sources and sampling

The analysis for this study was conducted based on data extracted from three consecutive Ethiopian demographic and health surveys namely EDHS 2005 (*n* = 130), EDHS 2011 (*n* = 309), and EDHS 2016 (*n* = 317). The use of pooled data helped in getting a larger sample size more powerful to detect existing significant associations. The surveys collect nationally representative data on various maternal and child health indicators, including feeding practices of infant and young children in 11 geographical administrations. In this study, data were used to describe the level, trend and determinants of ASF consumption among children 6–23 months of age. Analysis from the combined data was used to determine the predictors of ASF consumption. Specifically, the Household Recode (HR) and Kids Recode (KR) dataset types of the EDHSs were used to reach to the final sample for this analysis.

The EDHS uses a two-stage stratified cluster sampling technique to select households from each enumeration area (EA). The first stage, involves selecting clusters from a list of EAs. In the second stage, a complete listing and a subsequent random selection of households from each EA is done.

In the 2005 EDHS, a representative sample of approximately 14,500 households from 540 clusters was selected. The clusters were selected from the list of EA from the 1994 Population and Housing Census sample frame. The total number of households interviewed was 13,721, yielding a household response rate of 99%. Out of the total 14,717 eligible women, 14,070 were interviewed, yielding a response rate of 96%.

The 2011 EDHS was done on a representative sample of 17,817 households, which were selected using a stratified, two-stage cluster design from 624 EAs using a sampling frame by the 2007 Population and Housing Census, conducted by the CSA. Out of the 17,385 eligible women, complete interviews were conducted for 16,515, yielding a response rate of 95%.

In the 2016 EDHS, a total of 18,008 households were selected for the sample, of which 17,067 were available during data collection. Of the occupied households, 16,650 were successfully interviewed, yielding a response rate of 98%. Among the 16,583 eligible women for individual interviews in the interviewed households, only 15, 683 successful interviews were done, yielding a response rate of 95%.

Sine sampling weights are survey specific, re-adjustment needed to be done in the combined dataset. Thus, sampling weight was adjusted so that weighted observations would add up to a certain 1000,000.

Our analysis was done among last born living children aged 6–23 months, living in Tigrai region, and who live with the respondent. The detailed methodology including the sample design, the sampling framework and sample implementation, and response rates are well elaborated in the respective EDHS reports [21, 28, 29].

### Study variables

#### Outcome variable

Consumption of ASF is the outcome variable for this study. The questionnaire asked mothers/caretakers what types of foods the child had eaten in the 24 h before the survey. Consumption of any of milk, yogurt, cheese, eggs, fish, meat (including beef, poultry, pork, lamb, and any other meat not mentioned), and organ meats (e.g., liver) was considered as ASF consumption. The variable was dichotomized into “No ASF consumption” (coded “0”) and “ASF consumption” (coded “1”).

#### Explanatory variables

These variables were chosen based on the availability of information in the respective EDHS reports and their possible influence on consumption of ASF from previous studies. Variables such as age [18], residence [18], maternal education [30], household wealth [31], household ownership of assets [23], and ANC [32] were reported to influence ASF consumption by children.

Demographic variables included in this analysis were child age, child sex, and maternal age. Child age in months was taken as a continuous variable. Maternal age was categorized into three levels (15–24 years, 25–34 years, and 35–49 years). Besides, family size was grouped into < = 3, 4–6, and > = 7.

Socioeconomic factors included maternal/father’s education, maternal/father’s occupation, household ownership of assets and household wealth. Educational status was dichotomized into no formal education versus formal education. Occupational status was grouped as not working, agricultural works, and non-agricultural works. Household ownership of assets was calculated as an added score from a set of twelve assets including electricity, a watch or clock, a radio, a television, a mobile telephone, a non-mobile telephone, a refrigerator, a table, a chair, a bed with a mattress (cotton/sponge/spring), an electric mitad (a grill or cooktop used for preparing injera or bread), and a kerosene lamp/pressure lamp. Similarly, total number of livestock owned by a household was determined as a summed up variable from a set of livestock including cattle (no cattle data in the 2011 series), cow/bull, sheep, goat, camel, and chicken. Health service factors included frequency of ANC visit (grouped as none, 1–4 visits, or 4 and above visits) and place of delivery (grouped as home or health facility).

#### Statistical analysis

Data analysis was done using the survey “SVY” command of Stata version 14.0. In order to avoid the probability of including an observation that would happen due to the complex sampling design and to avoid potential bias sampling weights were used. Before data analysis started, data cleaning and selection of appropriate control variables were done. Descriptive statistics are presented using proportions and means.

Since our data was limited to only Tigrai region, we used the “subpopulation” command to tell Stata that all samples were still being used to calculate standard errors for confidence intervals. Besides, since the data have been combined from different waves of the EDHS, the strata and cluster variables were regenerated in the pooled dataset to ensure that they are unique to each EDHS data.

Initial analyses aimed at determining the magnitude of ASF consumption at each of the three EDHS to evaluate the trend over the 10 years period (2005–2016). Then, using the combined sample, binary logistic regression was used to point out predictors of ASF consumption. Variables that satisfied the cutoff point of *p-*value ≤ 0.25 in the bivariate model were entered into a multivariate logistic regression model. Then, Adjusted odds ratios (OR) and 95% confidence intervals (CI) were calculated using multivariate logistic regression. Finally, only those variables with statistical significance of *p* < 0.05 were considered as significant predictors of the dependent variable. Hosmer and Lemeshow goodness-of-fit test (at p > 0.05) was used to test model fitness. Multi-collinearity among independent variables was also assessed using variance inflation factor (VIF) considering a value of 10 as a cut off.

## Results

### Trends in sample characteristics

Our combined sample of three consecutive demographic and health surveys included 756 mother-child pairs. More than 80% of the study participants lived in rural areas. The percentages of mothers who attended formal education were 26.2%, 37%, 46.8%, in 2005, 2011, and 2016, respectively (p-trend = 0.004). The mean (SD) and median (IQR) child age in the combined sample were 14.3 (5) and 14 (8) months, respectively. Besides, the mean (SD) number of livestock decreased from 10.4 (7.9) in 2005 to 8.9 (8.4) in 2016 (Table 1).

**Table 1:** Trends in sample characteristics

Variable	EDHS 2005 (n=130) n (%)*	EDHS 2011 (n=309) n (%)*	EDHS 2016 (n=317) n (%)*	Pooled (n=756) n (%)*	p-trend
**Religion**					0.79
Orthodox	126 (97.5)	294 (95.0)	300 (94.0)	720 (94.9)	
Muslim	4 (2.5)	15 (5.0)	17 (6.0)	36 (4.1)	
**Place of residence**					0.18
Urban	6 (6.7)	43 (17.0)	64 (20.0)	113 (16.5)	
Rural	124 (93.3)	266 (83.0)	253 (80.0)	643 (83.5)	
**Child sex**					0.68
Male	64 (48.9)	157 (50.6)	150 (47.0)	371 (48.7)	
Female	66 (51.1)	152 (49.4)	167 (53.0)	385 (51.3)	
**Child age in months**					
Mean (SD)	15.0 (4.89)	14.0 (5.1)	14.1 (5.2)	14.3 (5.0)	
Median (IQR)	16 (6)	14 (8)	14 (9)	14 (8)	
**Age of respondent**					0.44
15-24	46 (35.7)	88 (29.3)	98 (32.8)	232 (32.0)	
25-34	58 (45.9)	145 (46.0)	133 (41.3)	336 (43.9)	
35-49	26 (18.4)	76 (24.7)	86 (25.9)	188 (24.1)	
**Maternal formal educational attainment**					0.004
No	100 (73.8)	198 (63.0)	167 (53.2)	465 (60.6)	
Yes	30 (26.2)	111 (37.0)	150 (46.8)	291 (39.4)	
**Maternal occupation**					0.001
Housewife	69 (55.0)	86 (28.8)	122 (40.9)	277 (38.8)	
Agricultural	47 (33.8)	149 (47.5)	110 (33.2)	307 (38.8)	
Non-agricultural	14 (11.2)	74 (23.7)	85 (25.9)	172 (22.4)	
**Partner formal educational attainment**					0.54
No	82 (61.6)	189 (62.7)	167 (52.9)	440 (58.4)	
Yes	48 (38.4)	120 (37.3)	125 (39.7)	291 (38.3)	
Missing	0	0	25 (7.4)	25 (3.3)	
**Partner occupation**					<0.001
No occupation	2 (1.9)	3 (1.0)	9 (2.9)	16 (2.1)	
Agricultural	117 (87.5)	228 (71.8)	134 (39.4)	478 (63.3)	
Non-agricultural	11 (10.6)	78 (27.2)	149 (50.3)	237 (31.3)	
Missing	0	0	25 (7.4)	25 (3.3)	
**Wealth index**					0.18
Poorest	47 (35.5)	91 (29.2)	94 (27.7)	232 (39.7)	
Poorer	32 (23.2)	68 (20.8)	66 (19.7)	166 (20.7)	
Middle	26 (19.3)	38 (11.4)	45 (14.2)	109 (14.1)	
Richer	18 (14.7)	51 (16.2)	31 (12.5)	100 (14.3)	
Richest	7 (7.3)	61 (22.4)	81 (25.9)	149 (21.2)	
**Number of household assets**					
Mean (SD)	1.59 (1.83)	2.57 (2.78)	3.1 (2.62)	2.64 (2.63)	
Median (IQR)	1.0 (2.0)	2 (4)	2 (4)	2 (3)	
**Total number of livestock**					
Mean (SD)	10.4 (7.9)	NA	9.1 (9.2)	8.9 (8.4)	
Median (IQR)	10 (9)		7 (13)	7 (9)	
**Ownership of land for agriculture**					<0.001
Does not own	23 (18.3)	74 (25.5)	128 (43.2)	225 (31.9)	
Own	107 (81.7)	235 (74.5)	189 (56.8)	531 (68.1)	

### Trend in consumption of animal source foods

**Fig. 1:**
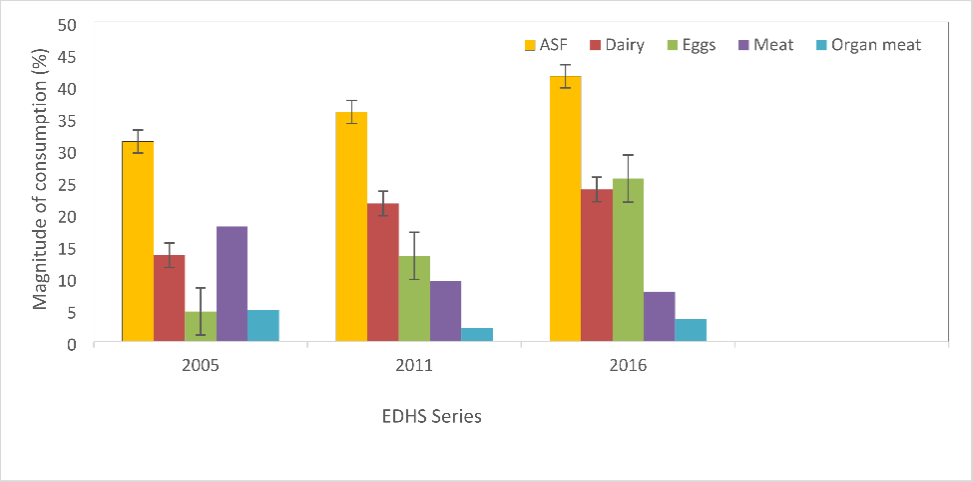
**Trends in consumption of animal source foods among 6–23 months old children in Tigrai across three EDHS series** ASF; Animal Source Foods EDHS; Ethiopian Demographic and Health Surveys

Overall consumption of animal source foods increased from 31.3% (95% CI 21.33, 43.31) in 2005 through 35.9% (95% CI 30.61, 41.58) in 2011 to 41.5% (95% CI 32.98, 50.5) in 2016. However, the difference was not significant (p-trend = 0.28) (Fig. 1).

### Predictors of animal source food consumption

In the bivariate analysis, eight variables met the criteria (p-value < 0.25) to be included in the multivariate analysis. These included place of residence, religion, family size, household wealth index, educational status of the respondent, total number of household assets, total number of household livestock, and child age. Finally, from the multivariable model, five variables were found to significantly predict ASF consumption (p-value < 0.05).

As compared to orthodox Christians, children from Muslim households had more than three (AOR = 3.1; 95% CI 1.39, 6.62) times higher likelihoods of consumption of ASF. Additionally, children from mothers who didn’t attend formal education were 33% (AOR = 0.67; 95% CI 0.47, 0.94) less likely to consume ASFs in contrast to those from mothers who attended formal education. For every month increase in the age of a child, a 9% (AOR = 1.09; 95% CI 1.05, 1.13) increment in the odds of ASF consumption was observed.

Besides, a unit increase in the total number of household assets was likely to lead to a 20% increase (AOR = 1.20; 95% CI 1.08, 1.34) in the odds of ASFs consumption. Furthermore, a unit increase in the total number of livestock was equivalent to a 2% (AOR = 1.02; 95% CI 1.01, 1.04) increase in the odds of ASF consumption (Table 2).

**Table 2:** Bivariate and multivariate analysis of determinants of ASF consumption among children aged 6-36 months in Tigrai, Ethiopia, n=878

Variables		ASF Consumption			COR (95%CI)	P-value	AOR(95%CI)	P-value
		No (n=480)	Yes (n=276)	Total				
Place of residence	Urban (Ref)	63	50	113	1	-	1	-
	Rural	417	226	643	0.75 (0.47,1.20)	0.23	1.17(0.44,3.13)	0.74
Religion	Orthodox (Ref)	465	255	720	1	-	1	-
	Muslim	15	21	36	2.6 (1.17,5.92)	0.03	3.1(1.39,6.62)	0.007^b^
Wealth index	Poorest	176	56	232	0.43(0.26,0.71)	0.001	1.05(0.39,2.782)	0.93
	Poorer	96	70	166	1.00 (0.59,1.72)	0.98	2.3(0.81,6.44)	0.11
	Middle	62	47	109	1.06(0.63,1.80)	0.81	2.2(0.82,5.85)	0.11
	Richer	61	39	100	0.95(0.54,1.66)	0.85	1.35(0.52,3.491)	0.53
	Richest (Ref)	85	64	149	1	-	1	-
Family size	<=3(Ref)	52	36	88	1	-	1	-
	4-6	260	151	411	0.75(0.49,1.13)	0.17	0.61(0.40,0.94)	0.06
	7 or above	168	89	257	0.67(0.43,1.08	0.10	0.64(0.39,1.07)	0.09
Respondent formal education	No	323	142	465	0.62(0.46,0.85)	0.003	0.67(0.47, 0.94)	0.02
	Yes (Ref)	157	134	291	1	-	1	-
Number of household assets	Mean (SD)	2.3 (2.5)	3.2 (2.7)	2.6 (2.6)	1.13 (1.06,1.214)	<0.001	1.20(1.08,1.34)	0.001^b^
Total Number of livestock	Mean (SD)	8.5 (8.2)	9.8 (8.7)	9.0 (8.4)	1.00 (0.98,1.02)	0.052	1.02(1.01,1.04)	0.039
Child age in months	Mean (SD)	14.0 (5.1)	14.7 (4.8)	14.3 (5.0)	1.07 (1.04,1.11)	<0.001	1.09(1.05,1.13)	<0.001^b^

## Discussion

This study was conducted to determine trends in and predictors of consumption of ASFs among 6–23 months old children from Tigrai, Ethiopia. The data used for this analysis was an extract from three consecutive EDHS databases.

Magnitude of ASF consumption increased from 31.3% (95% CI 21.33, 43.31) in 2005 through 35.9% (95% CI 30.61, 41.58) in 2011 to 41.5% (95% CI 32.98, 50.5) in 2016. This 10-percentage point increase in proportion of ASF consumption over the course of three back-to-back EDHSs could be the result of interventions that encouraged livestock production and local livestock markets, programs that raised awareness on nutritional benefits of ASFs, and community based projects that carried behavioral change components. For instance, a recently published work witnessed that provision of age-appropriate educational interventions for mothers increased the consumption of ASFs among children aged 6–23 months from rural communities of Tigrai [33].

Other studies done in Ethiopia have shown that children’s diet is mainly plant based [34]. Likewise, a food consumption survey done in Ethiopia reported a very low consumption levels of any flesh foods among children [35]. The role of ASFs in improving linear growth has already been stated. Consumption of eggs significantly improved growth in young children [36]. Besides, provision of children with ASFs including milk and meat, with even small quantities [37, has led to some improvements in physical growth and cognitive development [37, 38]. These attributes are due to the fact that animal proteins are of high quality and quantity [39] and that they contain plentiful asset of bioavailable micronutrients [40]. The quality of a protein shows highly digestibility, greater absorption rate, and timely availability for bodily processes [41].

Our study found out that the odds of ASF consumption were more than three times higher among children from Muslim families when compared to those from Orthodox-Christian households. This relatively very low level of consumption among children from Orthodox Christian households could be very much attributed to the very long periods of fasting throughout a year [42]. During these fasting periods, followers of the religion should remain denied of any food of animal origin. Therefore, the demand by households for or the supply of ASFs in the local markets may diminish eventually decreasing their availability for child consumption [43]. Additionally, consumption of ASFs among children living in such households would be limited because mothers/caregivers mightn’t be well inclined to use ASFs for cooking due to fear of contaminating their “fasting” foods [44]. Furthermore, such households consider slaughtering livestock during fasting periods a major violation of their religious doctrine [44]. Nonetheless, this was in conflict with another study that reported higher price but not fasting as a main constraint to egg consumption by young children who were from Orthodox households [33].

In this study, a month increase in the age of a child was associated with a 9% increase in the odds of ASF consumption. This finding is supported by a study done among children from four regions in Ethiopia that showed an 8% increment in the odds of consumption of ASFs with every 3-month increase in age [45]. As children get older, their requirement for nutrients is increased and it is more likely that they improve their feeding skills.

This study showed that children from mothers who didn’t attend formal education were 70% less likely to consume ASFs in contrast to their counterparts. A study from India reported that maternal formal education positively influenced child nutrition outcomes [46]. Likewise, another study from Cambodia described that maternal education indirectly improved child dietary and nutritional outcomes through immediate effects on other characteristics such as household wealth and maternal employment [47]. Although knowledge and attitude could be changed via tailored education campaigns, mothers/caregivers who attended formal education have better reading, writing, and other language skills that could increase their knowledge and understanding of child feeding and nutrition messages. In line with this, associations between caregivers’ knowledge and attitude on nutrition and children’s both frequency and diversity of ASF consumption has been published [48]. On a similar note, according to a qualitative study from Ghana, mothers/caregivers believed that children shouldn’t be offered much of ASFs as it would cause unrealistic taste preferences and expectations which are difficult to meet[49]. Notably, a unit increase in the number of livestock owned by a household was associated with a 2% increase in the odds of ASF consumption. Studies conducted in Sub Saharan Africa [50], Uganda [51], and Tanzania [52] reported similar findings. These evidences support that livestock ownership enhances the likelihood of families to serve ASFs to their children. Owning a small number of livestock would even increase consumption of ASFs [53]. Due to its benefits in enhancing consumption of ASFs and in increasing child dietary diversity [54], promoting livestock ownership should be one of the hotspots for organizations working on child nutrition.

Not differently, our study found out that a unit increase in the number of household assets led to a 17% increase in ASF consumption. Other studies showed similar findings [55, 56]. This may be explained by the fact that ownership of household assets and amenities may increase the likelihoods of, cooking, preparing, storing, and serving food including ASFs for consumption [57].

### Strengths and Limitations of the study

This study is the first of its kind in providing information on the trends in ASF consumption among children in Tigrai, a region with grave rate of child undernutrition. The use of sampling weights and cluster and strata variables minimized the risk of having biased estimates and standard errors. The use of sampling weights has entitled every household, rural or urban, an equal probability of inclusion in this study and ultimately reduced the risk of getting biased estimates. Besides, the use of strata and cluster variables would help in getting robust standard errors of the estimates. This study had some limitations. One, inclusion of information on possible predictor variables like household food insecurity status, perception of mothers/caregivers towards ASFs, cost of ASFs, women’s decision-making power on purchasing and feeding of ASFs, cultural rules, and others would have unfolded some other unseen relationships. Two, the information provided by this study is solely about consumption of any ASF and not about quantity (grams or servings) and/or frequency of consumption. Due to the cross-sectional nature of the study design, we can only talk about associations but not cause and effect relationships between the predictor and outcome variables.

## Conclusion

Consumption of ASFs showed a statistically non-significant increase over the three consecutive EDHS series. Religion, child age, respondent’s educational status, number of household assets, and number of livestock were found to be predictors of ASF consumption. Based on the findings of this study, child ASF consumption might be increased through pro-maternal education policies, programs with household asset increasing schemes, and pro-livestock projects. Our study also highlighted the need for considering religion as one important player when planning or undertaking ASF programs.

## Data Availability

Supporting data for the current study are available from the corresponding author upon a reasonable request. The EDHS data was retrieved from https://dhsprogram.com/data/available-datasets.cfm.

## Abbreviations

ANC: Ante-Natal Care
AOR: Adjusted Odds ratio
ASF: Animal Source Foods
CI: Confidence Interval
COR: Crude Odds Ratio
CSA: Central Statistical Agency
EA: Enumeration Areas
EDHS: Ethiopian Demographic and Health Surveys
IQR: Inter-Quartile range
LMIC: Lower and Middle Income Countries
OR: Odds Ratio
SD: Standard Deviation
SVY: Survey
VIF: Variance Inflation Factor
WHO: World Health Organization
